# Comparative effectiveness of congregation- versus clinic-based approach to prevention of mother-to-child HIV transmission: study protocol for a cluster randomized controlled trial

**DOI:** 10.1186/1748-5908-8-62

**Published:** 2013-06-08

**Authors:** Echezona E Ezeanolue, Michael C Obiefune, Wei Yang, Stephen K Obaro, Chinenye O Ezeanolue, Gbenga G Ogedegbe

**Affiliations:** 1 Department of Pediatrics, University of Nevada School of Medicine, 2040 West Charleston Boulevard, Las Vegas, NV, USA; 2 Prevention, Education, Treatment, Training and Research-Global Solutions-PeTR-GS, 7 Link Road, Independence Layout Enugu, Enugu State 400001, Nigeria; 3 School of Community Health Sciences, University of Nevada, Reno, Lombardi Building, Reno, NV 203MS 0274, USA; 4 University of Nebraska Medical Center, 982165 Nebraska Medical Center, Omaha, Nebraska, USA; 5 Sunrise Foundation, Plot 358 New GRA, Enugu, Nigeria; 6 School of Medicine, New York University, 550 1st Ave, New York, NY, USA

**Keywords:** HIV, Prevention of Mother-to-child HIV Transmission (PMTCT), Congregation-based Approaches, Community-based, Church-based, Comparative Effectiveness, Nigeria

## Abstract

**Background:**

A total of 22 priority countries have been identified by the WHO that account for 90% of pregnant women living with HIV. Nigeria is one of only 4 countries among the 22 with an HIV testing rate for pregnant women of less than 20%. Currently, most pregnant women must access a healthcare facility (HF) to be screened and receive available prevention of mother-to-child HIV transmission (PMTCT) interventions. Finding new approaches to increase HIV testing among pregnant women is necessary to realize the WHO/ President's Emergency Plan for AIDS Relief (PEPFAR) goal of eliminating new pediatric infections by 2015.

**Methods:**

This cluster randomized trial tests the comparative effectiveness of a congregation-based Healthy Beginning Initiative (HBI) versus a clinic-based approach on the rates of HIV testing and PMTCT completion among a cohort of church attending pregnant women. Recruitment occurs at the level of the churches and participants (in that order), while randomization occurs only at the church level. The trial is unblinded, and the churches are informed of their randomization group. Eligible participants, pregnant women attending study churches, are recruited during prayer sessions. HBI is delivered by trained community health nurses and church-based health advisors and provides free, integrated on-site laboratory tests (HIV plus hemoglobin, malaria, hepatitis B, sickle cell gene, syphilis) during a church-organized ‘baby shower.’ The baby shower includes refreshments, gifts exchange, and an educational game show testing participants’ knowledge of healthy pregnancy habits in addition to HIV acquisition modes, and effective PMTCT interventions. Baby receptions provide a contact point for follow-up after delivery. This approach was designed to reduce barriers to screening including knowledge, access, cost and stigma. The primary aim is to evaluate the effect of HBI on the HIV testing rate among pregnant women. The secondary aims are to evaluate the effect of HBI on the rate of HIV testing among male partners of pregnant women and the rate of PMTCT completion among HIV-infected pregnant women.

**Discussion:**

Results of this study will provide further understanding of the most effective strategies for increasing HIV testing among pregnant women in hard-to-reach communities.

**Trial Registration:**

Clinicaltrials.gov, NCT01795261

## Background

Globally, Sub-Saharan Africa (SSA) accounts for 76% of all women living with HIV and an estimated 90% of the 3.4 million children living with HIV. A total of 22 priority countries have been identified by the WHO that account for 90% of pregnant women living with HIV. Nigeria is one of only 4 countries among the 22 with an HIV testing rate for pregnant women of less than 20% [1,2]. Studies have shown that when HIV-infected women are treated with antiretroviral therapy and their infants receive antiretroviral (ARV) prophylaxis, mother-to-child transmission of HIV (MTCT) occurs in less than 1% of pregnancies [3-6]. Despite the availability of simple, less expensive and highly effective ARV regimens capable of being implemented in resource-limited settings, 75,000 Nigerian infants were infected in 2010 [1,7]. During that year, only 14% of pregnant women were tested for HIV, while 9% of pregnant women living with HIV received WHO recommended antiretroviral (ARV) therapy, and only 11% of HIV-exposed infants received ARV prophylaxis for prevention of mother-to-child HIV transmission (PMTCT) resulting in an estimated 75,000 HIV-infected infants in 2010 [1,8]. Currently, most pregnant women must access a healthcare facility (HF) to be screened and receive available PMTCT interventions. This clinic-based approach is challenging when only 35% of pregnant women deliver in an HF and only 2.9% of HFs have an established PMTCT program [8,9]. Finding new approaches to translate evidence-based interventions in PMTCT to sustainable community-based programs is necessary to realize the WHO/PEPFAR goal of eliminating new pediatric infections by 2015 [10].

Various reasons have been identified as barriers to HIV testing and PMTCT completion. Low perception of personal risk, poor access to testing sites, cost, confidentiality, and HIV-related stigma have all been identified as barriers to HIV testing [11-14]. Barriers to optimal PMTCT exist at the level of the patients and providers, but barriers at the health systems level appear to have more adverse impact on healthcare in general [15-17]. Most women in Nigeria do not access prenatal care early in pregnancy, and only 35% of pregnant women deliver in a health facility where just 2.9% have an established PMTCT program [9,18,19]. When community testing has been implemented to increase testing rate, the extensive attrition that takes place between HIV testing and treatment leads to people informed of their HIV-positive status failing to be adequately linked with appropriate services [20].

The late initiation of prenatal care results in missed opportunities for repeat testing, an important factor in PMTCT, as a significant proportion of pregnant women are in HIV-discordant relationships and testing rates are low among adult males, which could result in sero-conversions late in pregnancy [21-24]. Failure to disclose HIV sero-status is another barrier that hinders PMTCT completion, as studies have shown that women who do not disclose their sero-status to their male partners are less likely to complete interventions for PMTCT [25-27]. Male partner involvement has been shown to positively impact PMTCT, but asking or requiring pregnant women to recruit their male partners for HIV testing creates a barrier to partner testing, even though there is an increased willingness to test for HIV when pregnant women are tested at the same time with their male partners [28-31]. Despite these barriers, HIV testing exceeds 80% when pregnant women become aware of the effectiveness of interventions that assist in preventing MTCT and testing materials are available even in the presence of high anticipations of HIV/AIDS stigma [32-35].

Three viable and potentially cost-effective strategies for mitigating systems-level barriers to optimal testing and PMTCT completion include: decentralizing testing beyond health facilities [36,37], improving access to testing and treatment services, and identifying other delivery models that reduces loss to follow-up by linking testing sites to treatment centers [38-41].

Finding new approaches to translate evidence-based PMTCT programs to community-based settings is necessary if we are to realize the PEPFAR goal of 80% HIV screening rate among pregnant women by 2015 [10]. Fortunately, the government of Nigeria, in its 2010–2015 National Strategic Framework (NSF), identified reduction in new infections as a major goal [42]. It plans to increase access to quality HIV testing and ARV treatment for at least 80% of HIV-infected pregnant women. To achieve this, the framework calls for strengthening private sector engagement in expanding PMTCT interventions to community-based programs to complement existing clinic-based testing.

This study is one of few cluster randomized clinical trials of the effectiveness of congregation-based approach in hard-to-reach rural communities. We are reporting this method because it could serve as a model for other interventions aimed at improving maternal-child health outcomes in these communities.

### Study aims and hypothesis

The objective of this trial is to test the comparative effectiveness of a congregation-based Healthy Beginning Initiative (HBI) that promotes individual testing and utilizes the church network for subject tracking and retention versus a clinic-based approach in increasing HIV testing and PMTCT completion among 2,700 pregnant women and male partners attending 40 churches in Southeast Nigeria. We considered randomizing each individual patient, but the likelihood of contamination poses a threat to internal validity; thus, individual pregnant women will be nested within the church. We also considered a crossover design, but the possibility of withdrawing an intervention if it becomes effective would make this design problematic.

### Primary aim

The primary aim is to evaluate the effect of HBI on the HIV testing rate among pregnant women.

### Secondary aims

The secondary aims are to evaluate the effect of HBI on the rate of HIV testing among male partners of pregnant women and the rate of PMTCT completion among HIV-infected pregnant women.

### Hypotheses

We hypothesized that pregnant women randomized to the intervention group (IG) compared to those randomized to control group (CG) will have a higher rate of HIV testing, a higher rate of HIV testing among male partners, and a higher PMTCT completion rate among HIV-infected pregnant women. This study is one of few randomized trials to test the effectiveness of such a congregation-based approach in rural hard-to-reach communities.

## Methods

### Study design

**Figure 1:**
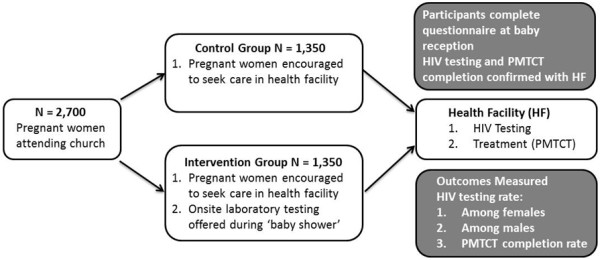
Overview of the healthy beginning initiative.

Using a two-arm cluster randomized trial design, we will evaluate the effect of a congregation-based HBI that provides free, integrated on-site laboratory tests during a church-organized baby shower as the IG versus clinic-based approach as the CG on the rate of HIV testing among pregnant women, their male partners, and the PMTCT completion rate among HIV-infected pregnant women. A total of 40 churches in Enugu State, south-east Nigeria, will be randomly assigned to either the IG (N = 20 churches) or the CG (N = 20 churches). A total of 2,700 pregnant women will be enrolled in this study, with about 8 to 16 pregnant women each month per church (depending on the size of the church) enrolled each month over a 5-month period. Participants in churches randomized to the CG will be referred to the closest healthcare facility for HIV testing and prenatal care. Participants in churches randomized to IG will receive health education and be offered an HIV test as part of an integrated laboratory test on-site at the church during a baby shower program. The primary outcome is difference in the rate of HIV testing between both groups. The secondary outcomes are the difference in the rate of HIV testing among male partners, and the PMTCT completion rate among HIV-infected pregnant women in both groups. Pregnant women will complete an investigator-administered questionnaire to collect information on HIV testing and PMTCT completion. Self-reported HIV testing and PMTCT completion will be confirmed with the healthcare facility for participants in churches randomized to the CG, and with on-site HIV testing data and the healthcare facility (PMTCT completion) for participants in churches randomized to the IG (Figure 1). Participants in churches randomized to CG will have two study visits: baseline (recruitment) and six to eight weeks after delivery. Participants in churches randomized to IG will have three study visits; baseline (recruitment), baby shower and six to eight weeks after delivery.

### Study setting and participants

We selected Enugu State as the site for the proposed study for three reasons: first, its population is culturally and ethnically related and predominately Christian, with church attendance approaching 90% [43,44]. Second, the overall state HIV sero-prevalence of 5.1% is close to the national average of 4.1% [8]. Third, the churches that we propose to use as study sites are widely distributed in Enugu and Oji-River areas, and represent variations in the prevalence rate across the state of 4% to 8% (6% average) [8]. Any self-identified pregnant woman attending any of the study sites is eligible to participate. Women are encouraged to participate with their male partners, but can still participate if the male partner chooses not to participate. The study was approved by the Institutional Review Board of the University of Nevada, Reno, and the Nigerian National Health Research Ethics Committee.

### Church recruitment and randomization

Recruitment occurred at the level of the churches and participants (in that order), while randomization occurred only at the church level. A total of 40 churches selected from four dioceses (the Anglican diocese of Enugu, the Catholic diocese of Enugu, the Anglican diocese of Oji-River, and the Catholic diocese of Agwu) were randomized to either the IG or the CG. All of the bishops who oversee churches in the proposed study areas had previously agreed to participate in this study and gave the study team access to speak to the priests under their dioceses. Randomization of churches occurred in 4 cohorts of 10 churches following random ranking. The churches were randomized in a 1:1 ratio to either the IG or CG. The sequence of randomization was generated by Dr Yang and kept in a sealed opaque envelop away from the study sites in accordance with CONSORT guidelines [45]. Once the sites had been recruited and baseline information on churches collected (*e.g.*, type, size of congregation), the sites were informed of their randomization group and assigned a code. Because of the nature of the intervention, it was impossible to blind the participants, community health nurses, lay health advisors, and study coordinators to the group assignment, but the test results cannot be influenced by the study coordinators.

### Staff recruitment and training

Prevention, education, treatment, Training and Research-Global Solutions (PeTR-GS), our local PEPFAR-supported partner working with the Sunrise Foundation, conducted training workshops for all study staff and church-based lay health advisors. They received training on the study protocol, including how to obtain informed consent, data collection forms, and confidentiality. Additionally, study staff received information on HIV counseling, delivering HIV test results and post-test counseling. Although priests are not involved in the active intervention, they also received information focused on basic HIV transmission, MTCT and PMTCT that will be useful should any participant identified as HIV-infected request counsel from the priest. Attempts were made to preferentially recruit individuals who have previously completed HIV training programs through PeTR-GS or other agencies to reduce the time spent in training. The training was a modified format used by PeTR-GS for national HIV counseling and testing training.

### Participant recruitment

Recruitment began following randomization of the churches. Each Sunday, the priest asks pregnant women and their male partners in the congregation to step to the altar for prayers. He prays for a healthy pregnancy, successful delivery, and encourages pregnant women to seek care at a health facility during their pregnancy. He introduces the concept of the Healthy Beginning Initiative and study team as a program supporting pregnant women in the congregation during pregnancy. Pregnant women and their male partners are encouraged to participate. Pregnant women who consent can participate in the study, even when the male partner is unavailable or chooses not to participate.

### Description of the intervention

**Figure 2:**
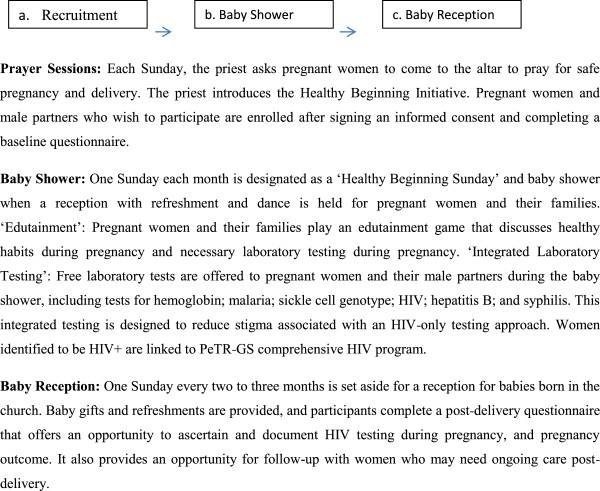
Summary of intervention activities.

Intervention is focused on removing system-level barriers to HIV testing and making tests available to pregnant women where they congregate as close to their home environment as possible. Conducted as part of a church-organized, family-oriented baby shower program that celebrates pregnancy and childbirth, the integrated approach to laboratory tests could potentially reduce the stigma associated with an HIV-only test approach (Figure 2).

### Description of the control condition/usual care

Participants in churches randomized to CG also follow the three steps of priest-directed prayer sessions, baby shower, and a church-organized baby reception. An edutainment game and free integrated on-site laboratory tests are not offered during the baby shower for this group. Pregnant women were encouraged to attend prenatal care through the health facilities. Participants also complete a post-delivery questionnaire. The research team maintains direct contact with health facilities to confirm HIV testing and PMTCT completion.

### Outcome measures

The primary outcome measure is HIV testing for pregnant women and pertains to both the cluster and individual participant level. Post-delivery questionnaires will be used to ascertain HIV testing during pregnancy. HIV testing during pregnancy will be confirmed through the integrated laboratory testing at churches randomized to IG. HIV testing among women in churches randomized to CG will be confirmed at the health facility where pregnant women reported prenatal care. The secondary outcomes are male partner HIV testing and PMTCT completion among HIV-infected women, and pertain to both the cluster and individual participant level.

### Sample size and power analysis

The proposed study is a two-arm cluster randomized controlled trial with the church as the unit of randomization. Thus, there are two important sample size estimates: N, the number of pregnant women; and K, the number of churches, with the pregnant women nested within the K churches. We reviewed the total annual infant baptisms in churches located in Enugu and Oji-River for 2010 to estimate the potential number of pregnant women. Our review indicated that there were 20,700 infant baptisms conducted in 2010. Further analysis showed that in 2010, Enugu and Oji-River each contained about 50 churches that had 200 infant baptisms and 40 churches that had at least 100 infant baptisms (Table 1).

**Table 1:** Infant baptisms in churches

**Enugu Local Government Area**	**Oji-River Local Government Area**
Catholic Churches: 9,300 infant baptisms	Catholic Churches: 9,000 infant baptisms
Anglican Churches: 1,400 infant baptisms	Anglican Churches: 1,000 infant baptisms

Power calculations were performed using the module ‘Inequality Tests for Two Proportions in a Cluster-Randomized Design’ in PASS 11, which implements methods of Donner and Klar [46]. That module approximates power for simple two-sample binomial tests for data collected in clusters with non-zero intra-cluster correlation (ICC). The Proc GLIMMIX analysis should have superior power since inclusion of covariates should reduce variability. For all three hypotheses, there will be 40 clusters (churches). For all three, there is an anticipated Control Group rate between 15% and 25% [1,8]. The usual recommendation is to take ICC between 0.002 and 0.05; ICC as high as 0.10 is considered quite high. The PASS module chooses a cluster size (number of individuals per cluster), which determines the total sample size as cluster size X, no. of clusters. Table 2 shows minimum detectable Intervention Group rates for various cluster sizes, Control Group rates, and ICC. The total sample size is driven by Secondary Hypothesis 2, where the CG rate should be around 0.20, and the intervention is expected to more than double the rate. From the preceding Table 2, it appears safe to target between cluster sizes 3 and 4, for a total sample size of 140 HIV-infected women. Using the average of 6% of pregnant women infected with HIV, and a possible 10% dropout rate (from our experience during scale up of community HIV testing), that would require (140/0.06)/0.9 or approximately 2,700 total pregnant women (1,350 per group). Clearly, any interesting increase in rate for the intervention group is detectable for such a large total sample size for Hypotheses 1 and 2 (Table 2).

**Table 2:** Minimum detectable intervention group rate (2-sided test, 5% significance level)

**Cluster size (total sample size)**	**Control group rate**								
	**0.15**			**0.20**			**0.25**		
	**ICC**			**ICC**			**ICC**		
	0.002	0.05	0.1	0.002	0.05	0.1	0.002	0.05	0.1
3 (120)	0.37	0.38	0.39	0.43	0.44	0.45	0.49	0.50	0.51
4 (160)	0.34	0.35	0.36	0.40	0.41	0.43	0.46	0.47	0.49
5 (200)	0.31	0.33	0.35	0.38	0.39	0.41	0.43	0.45	0.47
10 (400)	0.26	0.29	0.31	0.32	0.35	0.37	0.38	0.41	0.43
50 (2000)	0.20	0.24	0.27	0.25	0.30	0.33	0.31	0.36	0.39

Number of churches needed (K): We need to have 1,350 pregnant women in each arm of the study and follow them through pregnancy and up to six weeks post-delivery. Since pregnant women will be recruited at different stages of pregnancy, we will need to end recruitment five months after the study is open to recruitment and allow time to follow the last recruited pregnant women through nine months of pregnancy and up to six to eight weeks post-delivery (approximately eleven months). According to our review of infant baptisms in churches in our proposed study area, there were more than 50 churches with 200 infant baptisms per year (an average of 16 per month) and 20 churches with more than 100 infant baptisms per year (an average of 8 per month). We will need to recruit 15 of the large churches and 5 of the small churches in each arm to reach our sample size of 1,350 pregnant women in each arm over a 5-month period. Table 1 illustrates the flexibility we have with respect to both recruitment and attrition. For example, if our recruitment goal is an average of 16 pregnant women per month in a church with 200 infant baptisms per year, but it turns out we were only able to recruit an average of 12 participants per month, it means we will need to add and randomize 5 more churches (15 + 5 = 20) to maintain our level of statistical power. Note however, that our proposed primary analysis plan below is intent to treat (ITT), so we will analyze all participants who are randomized and who have completed initial testing even if they drop out later. Thus the above power analysis is conservative.

### Statistical methods

All three hypotheses test for differences in two binomial proportions at follow-up. Clustering effects must be incorporated in the analysis since data collected on parishioners within the same church could be expected to exhibit correlation, while data collected on parishioners in different churches should exhibit minimal correlation. Generalized linear mixed models as implemented in PROC GLIMMIX in SAS 9.3 [47] provide a rich set of tools for analyzing such data. Data will be analyzed with that procedure using a logit link function and the binomial distribution. These are multilevel models allowing incorporation of covariates and confounders for the individual (such as age, household income, education level, previous HIV testing, last menstrual period, marital status, employment status, male’s partners’ previous HIV test results, and situational/contextual factors) and cluster-level (*i.e.*, church) covariates and confounders such as size of church and congregation type (Anglican or Catholic). For all three hypotheses, a preliminary (and similar) analysis will model the probability that an individual declines participation; any significant predictors from that model will be used in the main analysis to reduce confounding. Statistical significance will be set as p-value <0.05 and tests will be 2-sided.

### Trial status

HBI began in December 2012 at 40 churches in Enugu State, Nigeria. To date, we have recruited 2,000 participants of the expected 5,400. Participant recruitment will be completed in August 2013, with each participant followed though pregnancy and up to eight weeks post-delivery.

## Discussion

### Challenges and limitations

In the first few months of implementing the study, we encountered several ‘real world challenges’ that resulted in some redesign of the protocol as originally conceived.

### Church recruitment

One of the most common barriers to recruitment of churches to participate in clinical trials involves the suspicion with which faith-based organization views the scientific community. We had anticipated that this might impact our recruitment, but to our surprise, we had such a willingness and insistence to participate from the priests that the 200 churches in the four dioceses all wanted to participate in the study. They saw the program as an evangelical tool to encourage and increase the number of males who traditionally do not attend church services regularly. Pregnant women in churches that were not initially selected were reportedly going to close by churches that were participating in the program. To avoid the potential impact of unstructured participation, we used a spoke-and-hub approach to include all the churches. Each initially selected church acts as a coordinating hub for surrounding churches that follows the randomization group of their coordinating church. This provided an organized method of expanding the number of churches to accommodate the high uptake without disrupting the randomization and follow-up schedule in the study. Baby shower receptions were held at the coordinating churches, while recruitment occurs at each individual church.

### Participant recruitment

Due to the high uptake of the program by churches and subsequent uptake by pregnant women, Sundays initially reserved for baby showers also became recruiting days. This initially overwhelmed the study team. Activities initially planned for an estimated 25 participating pregnant women and their male partners ended up being attended by 100 potential participants. The study team initially ran out of supplies of forms, mama packs, and blood sample collection kits. It took a couple of months before the study team was able to closely estimate attendance at baby shower programs. Initially, some pregnant women were turned away as consent forms were exhausted.

### Data collection

Although the questionnaire was constructed at a sixth grade reading level, administration of the questionnaire became very time consuming as we encountered a rather large number of individual participants who could not read English or the local language. The utilization of local church-based facilitators became very helpful in providing basic translation of information.

## Conclusions

The proposed study will test a congregation-based intervention that uses a family-centered, culturally-appropriate approach that relies on a widely distributed infrastructure (religious institutions). If successful, this approach could become a platform for multiple interventions aimed at improving the quality of maternal child health services especially in hard-to-reach communities.

## Abbreviations

HBI: Healthy beginning initiative; PMTCT: Prevention of mother-to-child HIV transmission.

## Competing interests

The authors declare that they have no actual or potential competing interests.

## Authors’ contributions

EE conceived of and designed the study and drafted the manuscript. MO participated in design and coordination of the study; GO participated in the design of study and manuscript preparation; WY programmed the randomization procedure, preliminary analysis and manuscript preparation; SO participated in study design and manuscript preparation; CE participated in study design and draft of manuscript. All authors read and approved the final manuscript.
